# The effect of altered dosage of a mutant allele of *Teosinte branched 1* (*tb1-ref*) on the root system of modern maize

**DOI:** 10.1186/1471-2156-15-23

**Published:** 2014-02-14

**Authors:** Amelie CM Gaudin, Sarah A McClymont, Sameh SM Soliman, Manish N Raizada

**Affiliations:** 1 Department of Plant Agriculture, University of Guelph, 50 Stone Road, Guelph, Ontario N1G 2W1, Canada

**Keywords:** Maize, *Zea*, Domestication, Teosinte, *Teosinte branched 1*, *Tb1*, Tiller, Root, Architecture, Lateral root, Crown root

## Abstract

**Background:**

There was ancient human selection on the wild progenitor of modern maize, Balsas teosinte, for decreased shoot branching (tillering), in order to allow more nutrients to be diverted to grain. Mechanistically, the decline in shoot tillering has been associated with selection for increased expression of the major domestication gene *Teosinte Branched 1* (*Tb1*) in shoot primordia. Therefore, TB1 has been defined as a repressor of shoot branching. It is known that plants respond to changes in shoot size by compensatory changes in root growth and architecture. However, it has not been reported whether altered TB1 expression affects any plant traits below ground. Previously, changes in dosage of a well-studied mutant allele of *Tb1* in modern maize, called *tb1-ref*, from one to two copies, was shown to increase tillering. As a result, plants with two copies of the *tb1-ref* allele have a larger shoot biomass than heterozygotes. Here we used aeroponics to phenotype the effects of *tb1-ref* copy number on maize roots at macro-, meso- and micro scales of development.

**Results:**

An increase in the *tb1-ref* copy number from one to two copies resulted in: (1) an increase in crown root number due to the cumulative initiation of crown roots from successive tillers; (2) higher density of first and second order lateral roots; and (3) reduced average lateral root length. The resulting increase in root system biomass in homozygous *tb1-ref* mutants balanced the increase in shoot biomass caused by enhanced tillering. These changes caused homozygous *tb1-ref* mutants of modern maize to more closely resemble its ancestor Balsas teosinte below ground.

**Conclusion:**

We conclude that a decrease in TB1 function in maize results in a larger root system, due to an increase in the number of crown roots and lateral roots. Given that decreased TB1 expression results in a more highly branched and larger shoot, the impact of TB1 below ground may be direct or indirect. We discuss the potential implications of these findings for whole plant coordination of biomass accumulation and maize domestication.

## Background

Evidence suggests that the domestication of maize (*Zea mays* ssp *mays*) began in the Balsas River valley of southwestern Mexico ~9,000 years ago from a wild grass relative known as Balsas teosinte *(Z. mays* ssp *parviglumis*) [1-7]. Descendant Balsas teosinte plants can still be found today in Mexico: they have a large shoot with multiple branches (tillers) each tipped with an inflorescence producing few seeds encapsulated by hard fruit cases. During maize domestication, the highly branched (tillered) shoot of ancestral teosinte was bred by ancient farmers into a crop with a single main stem, thus allocating more nutrients to seeds [8,9].

Significant research has been conducted on the genetic basis underlying the reduction in shoot tillering during maize domestication. These studies have implicated the transcription factor TEOSINTE BRANCHED1 (TB1) [10,11]. In single-stemmed genotypes of modern maize, tiller meristems responsible for shoot branching initiate but their outgrowth is repressed by TB1 [10-13]. *Tb1* is a member of the TCP Type II gene family involved in transcriptional regulation of cell cycle genes [14]. Mechanistically, it is hypothesized that TB1 may repress lateral bud outgrowth by binding to TCP Type II-specific binding sites in the promoters of cell-cycle genes, blocking their activation [15,16]. During domestication, ancient selection on the regulatory elements of *Tb1* resulted in increased *Tb1* expression in tiller meristems, leading to their constitutive repression [11,13,17-21]. *Tb1* is therefore considered a master shoot domestication locus and critical for the emergence of modern maize agriculture.

Given the dramatic *Tb1*-mediated changes in maize shoot architecture and size during domestication, it seems logical that there may have been balancing changes in root system size and morphology. Roots, which are otherwise metabolic sinks, are required for mechanical support of the shoot and for uptake of nutrients to support shoot growth; both requirements may have been reduced following domestication, at least during vegetative stages. Indeed, in an earlier study [22] we observed that Balsas teosinte and a modern maize inbred (W22) have a similar root:shoot biomass ratio despite thousands of years of crop selection, suggestive of selection inadvertently occurring at the whole plant level.

**Figure 1:**
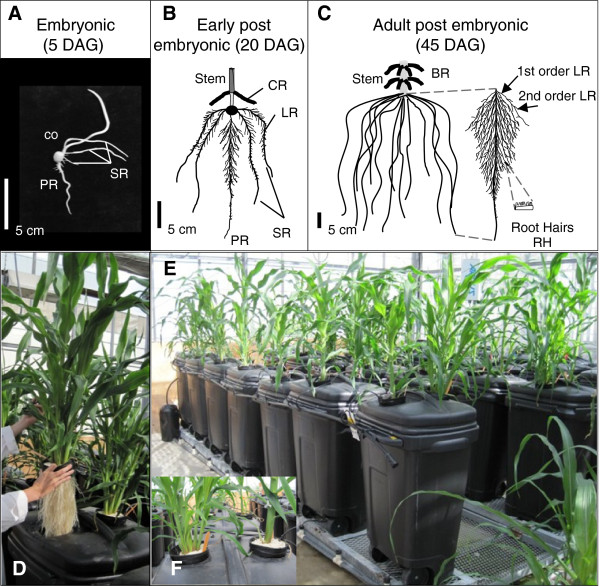
**Schematic representation of maize root system development and the aeroponics growth system used to facilitate root phenotyping. (A)** The primary root (PR) and seminal roots (SR) initiate from the embryo. **(B)** Lateral roots (LR) initiate from PR and SR, while crown roots (CR) initiate from the stem. **(C)** The root system in adult maize consists of a large number of crown roots and their LR, which undergo multiple orders of branching. The various root organs terminate in root hairs (RH). Structural brace roots (BR) initiating above ground are also shown. **(D-F)** The aeroponics growth system used in this study. **(D)** Picture showing the highly branched maize root system. **(E)** The custom built aeroponics chambers. **(F)** Modern maize *tb1-ref* plants (B73 background) (left) and inbred B73 (right) at 30 days after transplanting in the aeroponics system. Abbreviations: co = coleoptile; DAG = Days after germination.

The root system of maize is complex. At the seedling stage, maize relies on an embryonic root system consisting of a single primary root and a variable number of branched seminal roots [23] (Figure 1A). At adult stages, dozens of below- and above-ground shoot-borne roots, known as crown roots and brace roots, respectively, provide anchorage, nutrient uptake and transport to the shoot (Figure 1B). The thick and long crown roots initiate shorter and thinner first order lateral roots which further branch to give rise to even finer higher order lateral roots (Figure 1C). Lateral roots are the major contributors to root system length and nutrient uptake [24]. The epidermal surface of lateral roots and crown roots is covered with root hairs, which are single cell epidermal projections responsible for significant water and nutrient uptake [23].

Despite their importance, there has been no study concerning the impact of altered expression of *Tb1* on root systems in maize. Earlier studies have characterized a mutant allele of *Tb1* in modern maize called *tb1-ref*[25] which results in increased shoot branching [10,17]. These earlier studies demonstrated that the *tb1-ref* allele increased shoot branching in a dosage-dependent manner, with two copies of the allele resulting in more branches than heterozygotes [10,17]. In this study, we asked whether a change in dosage of *tb1-ref* has any effects on the root system of modern maize at the macro scale (crown roots), meso scale (lateral roots) and micro scale (root hairs). For our analysis, we compared one versus two copies of *tb1-ref,* which had previously been introgressed into modern maize inbred B73 [17], and were segregating here from a *tb1-ref/Tb1* × *tb1-ref/tb1-ref* testcross. We also analyzed B73 and Balsas teosinte as reference genotypes, the latter containing the ancestral *Tb1* allele.

There were two pre-requisites for this study. First, root phenotyping at adult stages was deemed critical, as differences in shoot branching between divergent *Tb1* different alleles become pronounced toward the end of the vegetative growth period. Second, since we hypothesized that TB1 may affect lateral root branching, a growth system was required that permitted non-destructive excavation of the fragile lateral roots and root hairs. For these reasons, we grew maize and teosinte plants in a customized aeroponics growth system (Figure 1D-F) where roots were suspended in the air and misted with a nutrient solution [26,27]. As we have demonstrated recently [24], aeroponics allows growth of maize to late vegetative stages, results in root system architecture that is similar to plants grown on solid substrate, and permits phenotyping of very large, intact root systems including fine lateral roots and root hairs.

Here we demonstrate that in modern maize, plants with two copies of the *tb1-ref* allele have a larger root system biomass than plants with a single copy, and that this biomass increase is associated with increased crown root and lateral root branching. Homozygous *tb1-ref* modern maize plants architecturally resemble ancestral Balsas teosinte both above and below ground. We discuss the potential implications of these findings for understanding the impact of *TB1* on whole plant coordination of biomass accumulation and maize domestication.

## Results

### The ancestral Balsas teosinte root system is highly branched, with a similar root:shoot biomass ratio as a modern maize inbred

**Figure 2:**
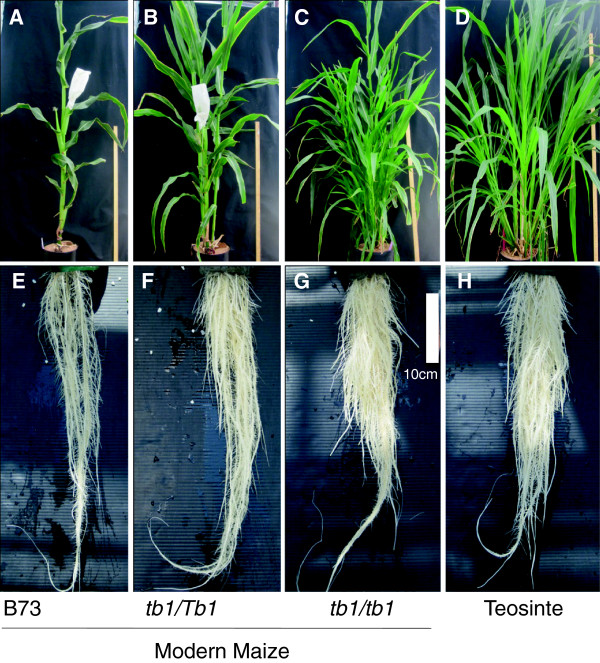
**Representative pictures of shoot and root morphologies of B73, *tb1-ref *and teosinte plants. (A-D)** Representative pictures of shoots (92 days after planting) of: **(A)** modern maize inbred B73, **(B)** heterozygous *tb1-ref* mutant *(tb1/Tb1,* B73 background), **(C)***tb1-ref* homozygous mutant (*tb1/tb1,* B73 background), and **(D)** Balsas teosinte. **(E-H)** Representative pictures of the root system at 35 days after transplanting (35 DAT) for **(E)** inbred B73, **(F)** heterozygous *tb1-ref* mutant *(tb1/Tb1*), **(G)***tb1-ref* homozygous mutant (*tb1/tb1*) and **(H)** Balsas teosinte.

Growth in aeroponics (Figure 1D-F) allowed us to compare the complete post-embryonic root systems of plants 35 days after transplanting (35 DAT). Balsas teosinte had a more tillered shoot but also a more bushy-looking root system compared to a modern maize inbred (B73) (Figure 2). As a result, compared to the modern inbred, the higher vegetative shoot weight of Balsas teosinte plants was balanced by its greater root biomass. Despite thousands of years of human selection separating these two genotypes, both the modern inbred and teosinte had statistically similar root:shoot biomass ratios (Table 1).

**Table 1:** **Dry biomass allocation, shoot and root traits in *tb1*-*ref *** **heterozygotes ( *tb1 */ *Tb1 *, B73 background) and homozygotes ( *tb1/tb1 *, B73 background) compared to modern maize inbred B73 and Balsas teosinte**

	**Tiller #**		**TRL (cm)**		**Dry weights (g/plant)**										**SRL (cm/g)**		**Leaf tips #**	
					**Total**		**Main stem**		**Tillers**		**Root**		**R/S ratio**					
*tb1/Tb1*	4 ± 1.0	a	76226 ± 5831	a	71.1 ± 4.8	a	36.3 ± 4.6	a	21.8 ± 4.3	a	12.9 ± 1.0	a	0.22 ± 0.03	a	5908.9 ± 510.2	a	45.6 ± 7.3	a
*tb1/tb1*	11.5 ± 1.6	b	119770 ± 8657	b	86.6 ± 7.9	b	27 ± 4.2	abc	42.6 ± 6.7	b	17.2 ± 1.5	b	0.24 ± 0.04	a	6963.4 ± 538.9	a	96 ± 11.6	b
B73	0	c	66900 ± 4321	a	63.2 ± 4.9	a	52.2 ± 4.1	b	0	a	11.1 ± 0.8	a	0.21 ± 0.02	a	6027 ± 522.7	a	14.5 ± 5.8	c
Teosinte	20.8 ± 1.1	d	124388 ± 93265	b	92.3 ± 9.8	b	19.7 ± 3.6	c	54.8 ± 4.8	c	18.7 ± 1.1	b	0.24 ± 0.03	a	6651.8 ± 567.4	a	126.5 ± 8.2	d
Genotype effect	**		*		*		**		*		*		NS		NS		*	

Notes.

-Values are Least Square Means from ANOVA ± standard errors (n = 6) at 35 days after transplanting.

-Means within a column followed by the same letter are not significantly different at α = 0.05. A mean value with multiple letters indicates that it is not significantly different at α = 0.05 from multiple other genotypes as indicated.

-(*) significant at α = 0.05, (**) significant at α = 0.01, NS = Not Significant.

-(#) = number, TRL = total root length, R/S = root to shoot biomass ratio, SRL = specific root length.

### Homozygous *tb1-ref* plants have a greater shoot and root biomass than heterozygotes

Segregating homozygous (*tb1-ref/tb1-ref*) and heterozygous plants (*Tb1/tb1-ref*) were distinguished using a diagnostic molecular marker (Additional file 1: Figure S1) and then phenotyped. As expected, in modern maize, the *tb1-ref* homozygous mutant mimicked the high shoot tillering phenotype of Balsas teosinte (Figure 2A-D; Table 1). Homozygous *tb1-ref* mutant plants showed a significant increase in shoot biomass compared to heterozygotes (Table 1). Modern maize plants that possessed two copies of *tb1-ref* were not significantly different than Balsas teosinte in terms of shoot biomass or tiller number (Table 1). Below ground, modern maize plants with two copies of *tb1-ref* had a significantly higher root biomass than heterozygotes (Table 1; Figure 2). Increased copy number of the *tb1-ref* allele caused the root biomass to increase sufficiently to balance its effect on increased tillering above ground, resulting in no significant change in the root:shoot biomass ratio (Table 1).

The specific root length (SRL, measured in cm/g) also increased in *tb1-ref* homozygotes compared to heterozygous plants (Table 1): the simplest interpretation is that this change was caused by a greater increase in lateral root length than crown root length in homozygotes, since lateral roots are very thin and light weight compared to crown roots.

### Homozygous *tb1-ref* plants have more crown roots (CR) than heterozygotes

**Figure 3:**
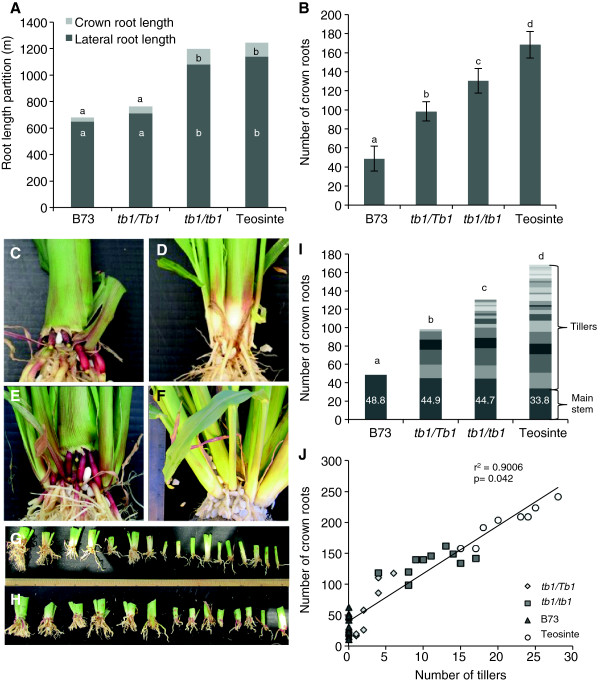
**Dosage effects of the *tb1-ref *allele on total root length and crown root traits at 35 DAT. (A)** Total root length and partition between crown roots and lateral roots. **(B)** Total number of crown roots. **(C-F)** Representative pictures of the crown region of: **(C)** modern maize inbred B73 (B73), **(D)***tb1-ref* heterozygous mutant (*tb1/Tb1*, B73 background), **(E)***tb1-ref* homozygous mutant *(tb1/tb1*, B73 background*)* and **(F)** Balsas teosinte. **(G, H)** Individual tillers and their associated crown roots initiating from the base of each stem in: **(G)***tb1-ref* homozygotes and **(H)** Balsas teosinte. **(I)** Crown root number partition between the main stem and successive tillers (n = 12). **(J)** Positive correlation between the number of crown roots and number of tillers across genotypes. Shown is the standard error of the mean estimate. Bars with the same letter are not significantly different at α = 0.05.

We investigated the underlying architecture of the heavier root systems of homozygous *tb1-ref* plants. Below ground, homozygous *tb1-ref* plants had an enlarged and bushier root system than heterozygotes, remarkably resembling the overall root system architecture of Balsas teosinte (Figure 2E-H) with similar total root length (Table 1). The total lengths of all crown roots (CR) and all measurable lateral roots (LR) were significantly greater in homozygous *tb1-ref* mutants compared to heterozygotes. The homozygous plants resembled ancestral Balsas teosinte for these traits (Figure 3A). Most significantly, the total number of CR, which are the thick and heavy backbone roots of maize, increased significantly in *tb1-ref* mutants compared to heterozygotes (Figure 3B).

### *tb1-ref* restores the ancestral developmental pattern of cumulative crown root initiation from successive tillers

We further investigated the CR phenotypes of *tb1-ref* mutants. We observed that CR initiate at the base of the main stem but also at the base of each tiller (Figure 3C-F). Therefore, we hypothesized that the *tb1*-*ref*-dependent increase in CR number was indirectly caused by its effect on increasing tiller number. To test this hypothesis, the association between tillers and CR was quantified. On *tb1-ref* modern maize plants, each shoot and its associated CR system were separated and measured individually. All mature tillers were observed to possess separate CR systems, similar to Balsas teosinte (Figure 3G-H; Additional file 2: Tables S1 and S2). The increase in CR number in homozygous versus heterozygous *tb1-ref* mutants was associated with increased tillering rather than more CR emerging from either the main stem or a subset of tillers (Figure 3I; Additional file 2: Tables S1 and S2). A similar positive association between successive tillering and crown rooting was observed in ancestral Balsas teosinte when compared to modern inbred B73 (Figure 3G-I; Additional file 2: Table S2). However, homozygous *tb1-ref* plants had fewer CR compared to its ancestor, perhaps due to a slower rate of tiller initiation in *tb1-ref* plants compared to Balsas teosinte (Additional file 2: Table S2). Consistent with these results, a direct positive correlation was observed between CR number and tiller number across genotypes (Figure 3J).

### Homozygous *tb1-ref* mutants have increased first order lateral root (LR) branching compared to heterozygotes

**Figure 4:**
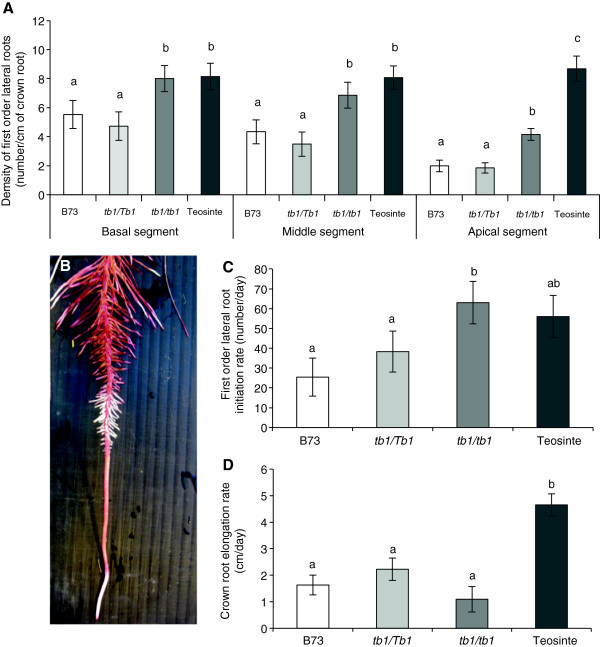
**Dosage effects of the *tb1-ref *allele on first order lateral roots. (A)** Density of first order lateral roots per segment of crown root for each of three crown root segments: top (basal), middle and bottom (apical). **(B)** Methodology used to measure root growth rate. The unstained tissue (white) represents new root growth at 24 hours after staining roots with neutral red. **(C)** Rate of first order lateral root initiation, and **(D)** crown root elongation over a 24 hour period beginning at 34 days after transplanting (DAT). At 15 DAT, synchronously initiating crown roots were labelled (one per plant) and all root traits were quantified 20 days later. Shown is the standard error of the mean estimate. Bars with the same letter are not significantly different at α = 0.05.

In maize, the CR initiate first order lateral roots (LR) which can further branch to form second order LR. First order LR branching initiates in the apical region of each CR above the elongation zone near the root tip; thus the most mature LR are located in the basal region closest to the soil surface. To quantify the effect of altered dosage of the *tb1-ref* allele on LR traits in modern maize, synchronously initiating CR were labelled (one per plant) at 15 days after transplanting (15 DAT). First order LR length and density were measured 20 days later on three equal segments above the elongation zone (see Methods). In all three CR segments, a significant increase was observed in the number of first order LR per cm of CR in homozygous *tb1-ref* plants compared to heterozygous *tb1-ref* plants (Figure 4A). In the oldest two root system segments, homozygous *tb1-ref* plants had a similar number of first order LR per crown root segment as Balsas teosinte (Figure 4A). These trends were similar when the total number of first order LR was expressed per CR segment (Table 2), indicating that the *tb1-ref* homozygous genotype led to an increase in the absolute number of first order LR on individual CR systems. To confirm this result, the number of newly initiated LR during a 24-hour period was scored at the same time as above. The branching zone of each CR was stained, and any unstained LR were scored as being newly initiated roots (Figure 4B-D). Using this method, homozygous *tb1-ref* plants were observed to have a higher rate of first order LR initiation than heterozygotes at 35 DAT (Figure 4C). To ensure that the higher rate of LR initiation was not an artefact of faster CR growth, the CR elongation rate over the same 24-hour period was simultaneously quantified. No difference was observed between the CR elongation rate in homozygous *tb1-ref* plants versus heterozygotes (Figure 4D). We conclude that plants with two copies of the *tb1-ref* allele have increased branching of first order LR compared to heterozygotes.

**Table 2:** Comparative measurements of the length and density of first and second order lateral roots per crown root segment

		**Per crown root segment**								**Per unit of crown root length (cm)**								**Per unit of 1**^**st **^**order LR**					
		**LR total length**				**LR number**				**LR total length**				**LR number**				**LR average length**				**LR number**	
**Segment**	**Genotype**	**1**^**st**^**order**		**2**^**nd **^**order**		**1**^**st **^**order**		**2**^**nd **^**order**		**1**^**st **^**order**		**2**^**nd **^**order**		**1**^**st **^**order**		**2**^**nd **^**order**		**1**^**st **^**order**		**2**^**nd **^**order**		**# 2**^**nd **^**order**	
Basal	*tb1/Tb1*	728.7	a	438.9	a	52.3	a	184.4	a	65.8	a	39.6	a	4.8	a	16.7	a	13.9	a	2.12	a	0.26	a
	*tb1/tb1*	803.3	a	468.5	ab	83.3	b	398.9	b	77.2	b	45.1	a	8.0	b	38.4	b	9.7	b	1.17	b	0.49	b
	B73	825.2	a	357.9	a	55.4	a	205.9	a	82.5	b	35.8	a	5.6	a	20.6	a	14.9	a	1.74	a	0.25	a
	Teosinte	950.4	a	503.6	b	106.7	b	504.3	b	72.5	b	38.4	a	8.2	b	38.5	b	8.9	b	1.00	b	0.53	b
Middle	*tb1/Tb1*	259.4	a	96.3	a	38.6	a	153.9	a	23.4	a	8.7	a	3.5	a	13.9	a	4.4	b	0.63	a	0.59	a
	*tb1/tb1*	289.2	a	93.9	a	71.3	b	282.3	ab	27.8	a	9.0	a	6.9	b	27.2	b	4.1	b	0.33	b	0.98	b
	B73	305.7	a	107.7	a	43.4	a	157.4	a	28.6	a	10.8	a	4.4	a	15.7	a	6.6	a	0.68	a	0.55	a
	Teosinte	490.1	b	113.3	a	105.7	b	408.7	b	39.4	b	8.6	a	8.1	b	31.2	b	4.9	b	0.28	b	0.79	b
Apical	*tb1/Tb1*	69.4	a	32.3	a	20.5	a	42.5	a	6.3	a	2.9	a	1.8	a	3.8	a	2.9	a	0.76	a	0.61	a
	*tb1/tb1*	79.6	a	29.7	a	47.3	b	31.1	a	6.9	a	2.6	a	4.2	b	2.7	a	1.6	a	0.26	a	0.39	b
	B73	41.8	a	46.4	a	17.9	a	38.6	a	4.6	a	5.2	a	1.9	a	4.3	a	2.3	a	0.30	a	0.93	a
	Teosinte	200.8	b	119.3	b	113.7	c	180.2	b	15.3	b	9.1	b	8.7	c	13.8	b	1.8	a	0.26	a	0.89	a

Notes

-Values are least square means from ANOVA ± standard errors (n = 12) at 35 days after transplanting.

-Means within a column followed by the same letter are not significantly different at α = 0.05. A mean value with multiple letters indicates that it is not significantly different at α = 0.05 from multiple other genotypes as indicated.

-LR = lateral root, WT = wild type, # = number.

### Homozygous *tb1-ref* mutants have increased second order lateral root (LR) branching compared to heterozygotes

**Figure 5:**
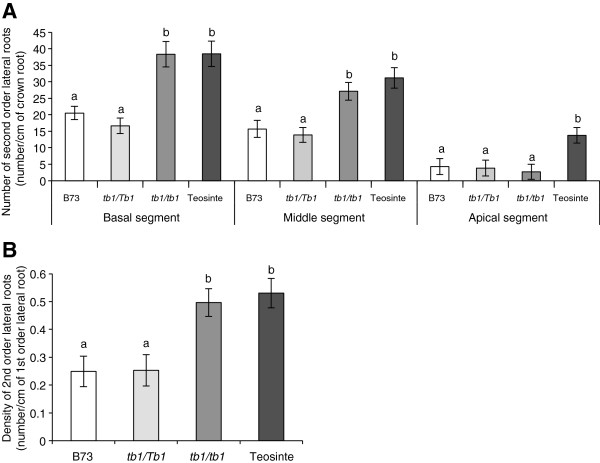
**Dosage effects of the *tb1-ref *allele on second order lateral roots. (A)** Total number of second order lateral roots per unit length of crown root for each of three crown root segments: top (basal), middle and bottom (apical). **(B)** Density of second order lateral roots per unit length of first order lateral root. Measurements were performed at 34 days after transplanting (DAT) on crown roots synchronously initiated at 15 DAT (n = 12). Shown is the standard error of the mean estimate. Bars with the same letter are not significantly different at α = 0.05.

On the two most mature CR segments, the basal and middle regions, homozygous *tb1-ref* plants initiated ~two-fold more total second order LR per cm of CR compared to *tb1-ref* heterozygotes. The homozygotes were similar to Balsas teosinte in the oldest two root system segments (Figure 5A; Table 2). A similar result was observed when the data was normalized by unit length of LR (Figure 5B; Table 2). No informative differences could be observed between homozygotes and heterozygotes in the segment closest to the CR tip, likely because the second order LR were in the process of initiating (Figure 5A; Table 2). We conclude that, along with first order LR branching, *tb1-ref* homozygous plants have an increased density of second order LR compared to heterozygotes.

Finally, the homozygous *tb1-ref* genotype showed reduced average lengths of both the first and second order LR by 31% and 45%, respectively, compared to heterozygotes on the most mature CR region (basal segment); the average lengths of LR of homozygotes were similar to Balsas teosinte (Table 2). Hence, even though homozygous *tb1-ref* plants had shorter LR compared to heterozygotes, the overall LR length per CR appeared to be the same as there was an increase in LR numbers.

### Altered dosage of *tb1-ref* does not affect root hairs (RH)

**Figure 6:**
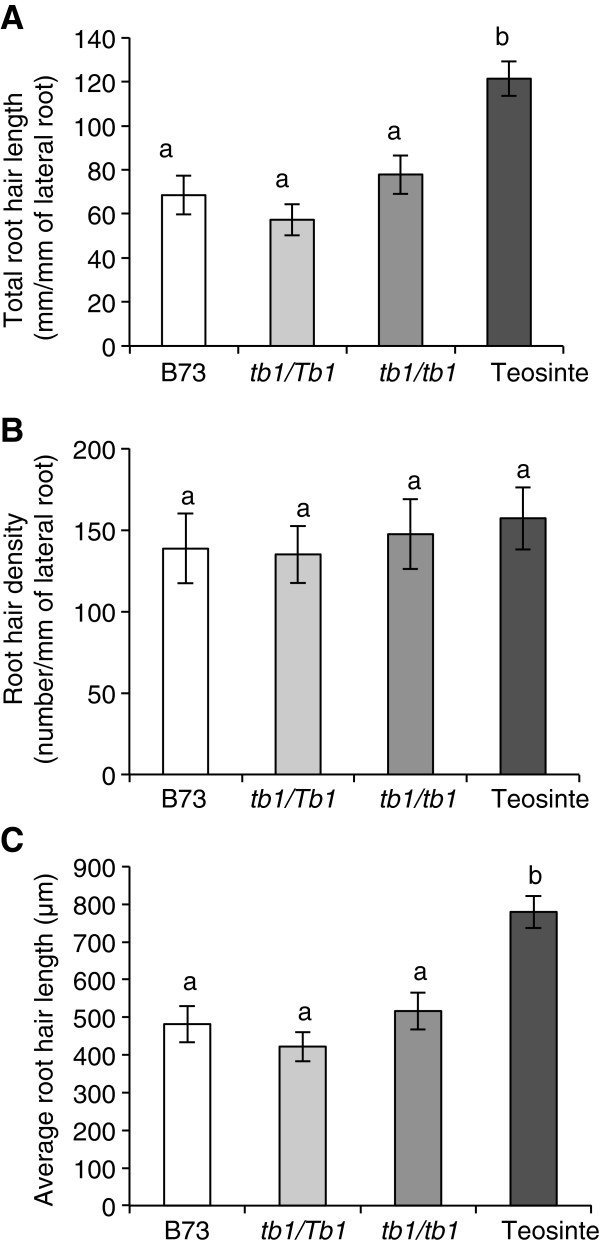
**Dosage effects of the *tb1-ref *allele on root hair traits at 35 DAT. (A)** Total root hair length per unit length of first order lateral roots. **(B)** Root hair initiation density per unit length of first order lateral roots. **(C)** Average root hair length. At 15 DAT, synchronously initiating crown roots were labelled (one per plant), and root hairs were scored 20 days later on lateral roots 5 cm away from the beginning of the branching zone on each crown root. Root hair traits were quantified using 192 digital images per genotype. Shown is the standard error of the mean estimate. Bars with the same letter are not significantly different at α = 0.05.

Unlike tillers, crown roots or lateral roots, all of which initiate from meristems, root hairs (RH) originate from differentiation of individual epidermal cells [28]. Altered dosage of the *tb1-ref* allele showed no significant effect on total RH length per unit of lateral root, average RH length or RH density (Figure 6). Total and average RH lengths were significantly higher in Balsas teosinte than all of the modern maize B73 genotypes, consistent with independent data comparing Balsas teosinte with another modern maize inbred line (W22) [22].

## Discussion

**Figure 7:**
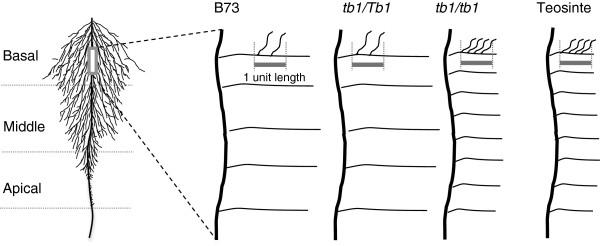
**Summary of the dosage effects of the *tb1-ref *allele on lateral root branching and length.** Shown is a schematic representation of a single crown root with its first and second order lateral roots.

Earlier studies demonstrated that reduced-function alleles of the major maize shoot domestication locus *Tb1* result in an increase in biomass and axillary branching of the shoot [10,11,25]. TB1 orthologs have also been shown to have conserved above-ground functions in Arabidopsis [29], sorghum [30], rice [31], wheat [32] and barley [33]. However, the below ground impact of mutations at the *Tb1* locus or its orthologs, had not been reported previously. In this study, using aeroponics, we observed that in *tb1-ref* mutants, the increase in shoot biomass was balanced by a corresponding increase in root biomass. Increasing the *tb1-ref* copy number from one to two copies altered root architecture at the macro and meso scales of root development, resulting in: (1) an increase in crown root number due to the cumulative initiation of crown roots from successive tillers; (2) higher density of first and second order lateral roots; and (3) reduced average lateral root length (Figure 7). These changes caused homozygous *tb1-ref* mutants of modern maize to resemble the root system of its ancestor Balsas teosinte (Figure 7).

A limitation of this study is that we were not able to compare *tb1-ref* (B73) plants to a true wild-type, as the *tb1-ref* seeds that we received did not segregate wild-types because it was generated from a testcross (*tb1-ref/tb1-ref* x *Tb1-ref/tb1-ref*). Therefore, we limited our conclusions to the effects of one versus two copies of the *tb1-ref* allele which were segregating 1:1 in the same genetic background. For reference, we also characterized the root system of a standard B73 line, but we have not made direct comparisons out of caution that the two B73 backgrounds may be genetically or epigenetically different. Another limitation of this study is that though we focused on a well characterized mutant allele of *tb1*[10,17], we did not characterize any other *Tb1* alleles within modern maize. However, the variation observed in shoot tiller number and crown root number within and between the two comparison genotypes (*tb1/tb1* versus *Tb1/tb1*) clearly demonstrates that these populations are distinct (Additional file 1: Figure S2), and does not lead us to suspect the existence of a major segregating genetic modifier in the background that affects shoot or root branching; in any case, such a modifier should be equivalently segregating in both *tb1-ref* homozygotes and heterozygotes. We cannot rule out the possibility that a mutation in a tightly linked locus or distant regulatory element modulates the phenotypes observed here.

### Maize maintains homeostasis of the root:shoot biomass ratio despite mutation at *Tb1*

The root:shoot biomass ratio is considered a fundamental physiological and genetic measurement of how plants acclimate and adapt to the environment, respectively [24,34]. Despite having an enlarged shoot, Balsas teosinte plants were calculated as having a similar root:shoot biomass ratio as two modern maize inbreds (Table 1) [22]. This result is consistent with data from wheat and barley, in which no significant differences were observed in the root:shoot biomass ratio between modern cultivars and their wild ancestors [35].

What is mechanistically responsible for maintaining homeostasis in the root:shoot biomass ratio despite thousands of years of human selection on crop shoots? Similar to the teosinte versus modern inbred comparison, we found that homozygous *tb1-ref* mutants had more shoot tillers and crown root systems than heterozygotes. One possible explanation was that homozygous *tb1-ref* plants had more crown roots per (main) stem. Consistent with this hypothesis, in earlier studies involving modern maize, plasticity in crown root number in response to shading [36,37] or low nitrogen stress [24] was shown to occur without corresponding changes in tillering as these plants maintained a single stem. Furthermore, in rice, initiation of tillers and crown roots was shown to be uncoupled: in a knockout of the polar auxin efflux carrier OsPIN1, there was an increase in tiller number but a reduction in adventitious root number [38]. However, in the current study, we observed that the majority of the extra crown root systems in homozygous *tb1-ref* plants, compared to heterozygotes, initiated from the base of the (extra) tillers rather than from the main stem (Additional file 2: Tables S1 and S2; Figure 3C-J). Compared to inbred B73, the extra crown root systems in Balsas teosinte also initiated from the extra tillers (Additional file 2: Table S2; Figure 3C-J). There was a positive correlation between tiller number and crown root number across genotypes (Figure 3J). Therefore, the simplest explanation for root:shoot homeostasis in maize (and perhaps other crops) over thousands of years is that as the tiller number was reduced during domestication, there were successively fewer crown root systems because these initiate at the base of tillers. With respect to the impact of mutation at the *Tb1* locus, plants appear to have an auto-regulated developmental mechanism that adjusts root construction with any major change in the demand for nutrients caused by altered *Tb1-*dependent tillering.

Tillering and crown rooting appear to be similarly correlated in other species. For example, in sorghum, the ortholog of TB1 (*SbTB1*) was shown to be involved in the shade-dependent decline in tillering [39], a stress which decreases crown root number in maize [36,37]. In other cereals, Green Revolution breeding for dwarfism increased both tiller number and crown root number in wheat, barley and rice [40-43]. A single locus (*ari.e.*GP) was implicated in regulating both tiller number and crown root number in barley [44]. In wheat, decreases in crown root and tiller number were coordinated in response to reduced Red:Far Red light (shading) [45,46]. In a dicot, petunia, a defect in strigolactone synthesis increased both shoot branching and late-developing adventitious roots [47,48].

One important caveat to the above interpretation is that we do not know how mutations at the *Tb1* locus affect the root:shoot biomass ratio at reproductive stages of development when grain demand for nutrients may affect biomass partitioning to roots versus shoots. Due to the exponential increase in microchamber space and nutrients required to grow mature maize plants in aeroponics, this study was terminated at late vegetative stages. In studies concerning wheat and barley, the observed increase in seed weight following domestication was found to strongly correlate with a larger embryonic root system [49-51].

### Is the effect of *TB1* on lateral root branching direct or indirect?

We observed that plants with two copies of the *tb1-ref* allele had an increased density of first order and second order lateral roots (LR) (Table 2). Mechanistically, a key first step in lateral root primordia initiation is reactivation of cell division involving cyclin genes within the pericycle layer adjacent to xylem pole cells [52-54]. Altered cyclin regulation has been shown to affect the density of lateral roots [52]. In the shoot, it is proposed that TB1 inhibits tiller meristem outgrowth by binding to, and blocking activation of, cell cycle promoters [15,16]. Hence, one model is that the *tb1-ref* allele might directly increase lateral root density by preventing TB1-mediated repression of cell cycle genes involved in lateral root primordia (LRP) initiation.

Another possibility, however, is that the lateral root phenotype of *tb1-ref* plants may also have an indirect physiological cause, for example due to an increase in demand by the larger shoot for nutrients or alterations in phytohormone gradients associated with morphological changes. Consistent with an indirect physiological mechanism, we recently demonstrated that an increased density of second order lateral roots is an important response to low nitrogen in modern maize [24].

### Is there an adaptive advantage to shorter lateral roots in *tb1-ref* homozygous plants?

As noted above, the increase in lateral root number per segment of crown root in plants with more tillers (*tb1-ref* homozygotes) (Table 2) might permit a higher rate of nutrient uptake to support a larger shoot system. The adaptive advantage of homozygous *tb1-ref* plants showing a concomitant decrease in average lateral root length (Table 2) is less clear. One possibility is that the decline in average lateral root length occurs to metabolically compensate for the dramatic doubling in lateral root number, as the root system is an energy sink. This hypothesis is supported by the observation that the normalized total length of the lateral root system was not significantly or dramatically greater when the *tb1-ref* copy number was increased (Table 2). It might be that a more branched root system is the most energetically efficient means of adding mechanical stability to support a larger shoot by gripping the soil. Alternatively, an increase in lateral root number might be more adaptive than increasing the lateral root length in terms of reducing physiological bottlenecks for nutrient uptake, because the former increases the number of lateral root to crown root junctions for nutrient unloading into the thicker vascular system of crown roots.

## Conclusions

Future experiments are needed to quantify and/or localize TB1 protein or its orthologs in lateral root primordia cells including under different environmental stresses. Above ground, it has been proposed that *Tb1* expression in teosinte is environmentally regulated, whereas in modern maize it is more constitutively expressed to repress tiller outgrowth [55]. Correlating DNA sequence polymorphisms in diverse *Tb1* alleles from teosintes and modern maize [56] with their corresponding root phenotypes, may also help to clarify the role of *Tb1* in root branching including any role it may have played below ground during maize domestication. In particular, novel alleles of *Tb1* which can unlink root branch phenotypes from tillering would be particularly informative in maize or other species.

## Methods

### Plant materials

Maize inbred line B73 was obtained from the Maize Genetic Cooperative Stock Center (Accession NSL 30053, Lot 04ncai02, USDA, North Central Regional Plant Introduction Station). *Zea mays* ssp. *parviglumis* (Balsas teosinte) seeds were obtained from CIMMYT, Mexico (ID 9477). Balsas teosinte originates from the Central Balsas River Valley in Southwest Mexico and is thought to be the closest wild direct ancestor of modern maize [1,7]. Homozygous and heterozygous *tb1-ref* mutants were in a B73 background [17]. This material was previously generated by introgressing the *tb1-ref* allele for at least five generations into a B73 inbred obtained from Pioneer HiBred International which was then self-pollinated [17], and maintained by the laboratory of Paula McSteen (University of Missouri). The seeds used here were the progeny of a testcross (*tb1-ref/tb1-ref* x *Tb1/tb1-ref*) and hence were segregating 1:1, with no wild-types. Segregating *tb1-ref* alleles were distinguished at Guelph using marker umc1082 which is tightly linked to the *tb1-ref* allele (chromosome 1L, Bin 1.09) using primers, rev: 5′-GCCTGCATAGAGAGGTGGTATGAT-3′ and fwd: 5′ CCGACCATGCATAAGGTCTAGG-3′ with standard amplification conditions (Additional file 1: Figure S1).

### Plant growth system

Maize plants were grown in a custom-made aeroponics growth system (Figure 1D-F). In aeroponics, plants are grown by misting roots, suspended in the air, with a nutrient solution in a closed loop. Pairs of seedlings were transplanted into containers suspended on top of 133 L black micro-chambers containing internal microjets that were connected to a nutrient solution tank; the solution was replaced weekly. Four independent but identical aeroponics systems were constructed side-by-side. For each system, a 100 L nutrient solution fed 12 plants distributed amongst 6 barrels. Aeroponics permitted non-destructive sampling of the large post-embryonic root system of maize. Details of our aeroponics system and its construction have been previously described [24].

### Growth conditions and experimental design

Seeds were surface sterilized using 20% bleach with 0.05% Tween 20 for 5 min, and washed twice for 10 min each with water. Teosinte fruit cases were cut closest to the radicle with a nail clipper to improve the homogeneity of germination. Seedlings were germinated in the dark with dH_2_O-soaked filter paper with 1 mL of Maxim XL™ fungicide (Syngenta, USA). Uniformly germinated seedlings were transferred to the aeroponics growth system in a glass greenhouse under a mixture of high pressure sodium and metal halide lamps (800 μmol m^-2^ s^-1^, at pot level), 16 h photoperiod, and 28°C day /20°C night regime, during the summer of 2009, in the Crop Science Greenhouse Facility, University of Guelph. Six plants per genotype were grown for 35 days (12 leaf tips on average for B73) in a randomized block design. The experiment was repeated two times (n = 12).

The nutrient solution contained: 6 mM Ca(NO_3_)_2_, 4 mM NH_4_NO_3,_ 1 mM MgSO_4_, 0.1 mM, K_2_SO_4_, 1 mM KCl, 2 mM KH_2_PO_4_, 0.04 mM H_3_BO_3_, 0.02 mM MnSO_4_, 0.7 μM ZnSO_4_, 0.3 μM CuSO_4_, 0.5 μM NH_4_Mo_7_O_24_, 1 mM Fe-DTPA. Seven days after planting (3 leaf tip stage for B73), 3 g of EDTA-chelated micronutrient mix (Plant-Prod #7906B7B, Plant Products Co., Canada) was added per 100 L of the above solution to obtain a final concentration of 2.1 ppm Fe (5% EDTA chelated and 2% DTPA chelated), 0.6 ppm Mn, 0.12 ppm Zn, 0.03 ppm Cu, 0.39 ppm B and 0.018 ppm Mo. On a daily basis, the solution was maintained in the pH range of 5.7-6.3.

### Shoot measurements

Given the dramatic differences in plant development between Balsas teosinte, the B73 inbred and the *tb1-ref* mutants, all comparisons were performed at the same age rather than phenological stage. At 35 days after transplanting (DAT), shoots were analyzed for biomass partition between tillers, stems and leaves after 48 hours of drying at 82˚C.

### Macro-scale root measurements

During plant development, the total numbers of crown roots and tillers (as applicable) were recorded every 5 days. Root systems were harvested at 35 days after transplanting (DAT), weighed and flat-stored in trays containing 50% ethanol at -20°C. Twelve hours prior to root scanning, roots were thawed, floated in water in 30 × 42 cm transparent plastic trays, and scanned using a Large Area scanner (LA2400, Hewlett Packard, USA). Root traits were quantified using WinRhizo software root diameter analysis (Version PRO 2007, Regent Instruments Inc., Canada). Scans were analyzed for total root length per plant (TRL), and image analyzer was set up to measure length per diameter class allowing analysis of lateral roots (LR < 0.2 mm) and crown roots (CR > 0.5 mm) separately. Brace roots were excluded. The number of crown roots was scored by counting their initiation in the crown region. The masses of roots were then taken following drying at 82˚C for 48 hours.

### Meso-scale root measurements

At 15 days after transplanting (DAT) one newly initiated crown root on each plant was labelled by carefully tying a thread around it. At 35 DAT, these labelled roots were harvested, flat stored in 50% ethanol at -20°C in a transparent plastic bag, and later scanned. Because the first and second order lateral roots were not differentiated accurately by the WinRhizo software, we traced each lateral root order on different transparent sheets and then analyzed the tracings in WinRhizo. As crown roots varied in length, lateral roots were measured on each crown root as follows: each crown root was divided into 3 equal segments above the branching zone, and then each segment was further divided into 3 sub-segments. Within each segment, the middle sub-segment, representing 1/9 of each crown root, was directly measured and then multiplied by three to give the score for that segment.

### Analysis of lateral branching and crown root growth rate

The methodology used here was previously described [24]. At 15 DAT, synchronously initiating crown roots were labelled as above (one per plant, different roots than above). Twenty days later, each crown root tip was stained with 1 mM neutral red dye (pH 7) for 10 min and washed in dH_2_O for 5 min before being transferred back to the aeroponics growth system. Pictures were taken 24 hours after staining: the length of the non-dyed crown root tip and unstained lateral roots were used to quantify crown root elongation and lateral root initiation, respectively, during the 24 hour period. We previously tested various dye concentrations to ensure that staining had no effect on root growth (data not shown).

### Root hair (RH) (micro-scale) measurements

Four first order lateral roots were removed in a region beginning 5 cm distal to the crown root elongation zone from the 20 DAT crown roots described above. Samples were stored in deionised water at 4°C. Lateral roots were stained with 0.1% Trypan blue solution for 2 min, followed by washing with distilled H_2_0 for 1 min. RH density (RHD) was measured by counting root hairs on the full semi-circular plane of a 2 mm lateral root segment under a light microscope (Zeiss, 100X). This measurement was then multiplied by two for an estimate of the total root hair number per lateral root segment.

Root hair (RH) lengths were measured using a light microscope (Leica MZ8) with a 1/0.01 micrometer; four images per lateral root were taken using Northern Eclipse software (v5.0, Empix Imaging Inc, Canada). Images were exported to *ImageJ* software (V1.40g, NIH, USA). The scale in the Analyze function was set to 37 pixels/100 μm based on the microscale.

Total RH length per 100 microns of lateral root (TRHL) was quantified by digitally tracing every RH in ImageJ; only protruding root hairs in side-profile were traced. Additional methodological details have been described earlier [24]. The root hairs were traced from a total of 192 digital images per genotype to ensure robust measurements; each image contained ~200 root hairs.

### Statistical analysis

Statistical analyses were performed using the MIXED procedure of the SAS statistical software package (Version 9.1, Statistical Analysis System, SAS Institute, USA). Residuals were tested for normality using the Shapiro Wilk normality test; Lund’s test was used to identify and remove outliers. Unbalanced Two-Way Analysis of Variance and partition were calculated to determine differences between genotypes using the F-test with a Type I error alpha = 0.05. Tukey’s HSD test was used for multiple comparisons of means. Linear regression analysis of crown root number and tiller number was performed using SAS Linear Regression (PROC REG Procedure).

## Abbreviations

CR: Crown root; LR: Lateral root; RH: Root hair

## Competing interests

The authors have declared that no competing interests exist.

## Authors’ contributions

ACMG conducted all experiments with assistance from SAM and SSMS, ACMG and MNR designed the study and wrote the paper. All authors discussed the results and commented on the manuscript. All authors read and approved the final manuscript.

## Supplementary Material

Additional file 1: Figure S1. Example of *tb1-ref* allele genotyping using the umc1082 diagnostic PCR molecular marker. Shown is a 2% agarose gel performed on maize seedlings that were subsequently subjected to morphometric analysis in the greenhouse. **Figure S2.** Phenotypic variation within and between populations of *tb1-ref* homozygous and heterozygous plants at 35 days after transplanting for (A) the total number of crown roots per plant and (B) the total number of shoot tillers per plant. The range of values demonstrates that the two genotypes had distinct phenotypes associated with altered *tb1-ref* allele dosage, despite hypothetical genetic modifiers that may or may not have been segregating in the background (n=12). Click here for file

Additional file 2: Table S1. Comparisons of tiller initiation date (days after transplanting) in *tb1-ref* heterozygotes (*tb1/Tb1*, B73 background) and homozygotes (*tb1/tb1*, B73 background) compared to modern maize inbred B73 and Balsas teosinte. **Table S2.** Comparisons of numbers of associated crown roots initiating from the base of each stem or tiller in *tb1-ref* heterozygotes (*tb1/Tb1*, B73 background) and homozygotes (*tb1/tb1*, B73 background) compared to modern maize inbred B73 and Balsas teosinte. Click here for file
