# Supplementary material for: The effect of altered dosage of a mutant allele of Teosinte branched 1 (tb1-ref) on the root system of modern maize
Source: BMC Genet. 2014 Feb 14;15:23. doi: 10.1186/1471-2156-15-23 (PMC3930895; doi:10.1186/1471-2156-15-23)
Supplement: Additional file 1: Figure S1. — Example of tb1-ref allele genotyping using the umc1082 diagnostic PCR molecular marker. Shown is a 2% agarose gel performed on maize seedlings that were subsequently subjected to morphometric analysis in the greenhouse. Figure S2. Phenotypic variation within and between populations of tb1-ref homozygous and heterozygous plants at 35 days after transplanting for (A) the total number of crown roots per plant and (B) the total number of shoot tillers per plant. The range of values demonstrates that the two genotypes had distinct phenotypes associated with altered tb1-ref allele dosage, despite hypothetical genetic modifiers that may or may not have been segregating in the background (n=12). [file 1471-2156-15-23-S1.pdf]

Figure S1

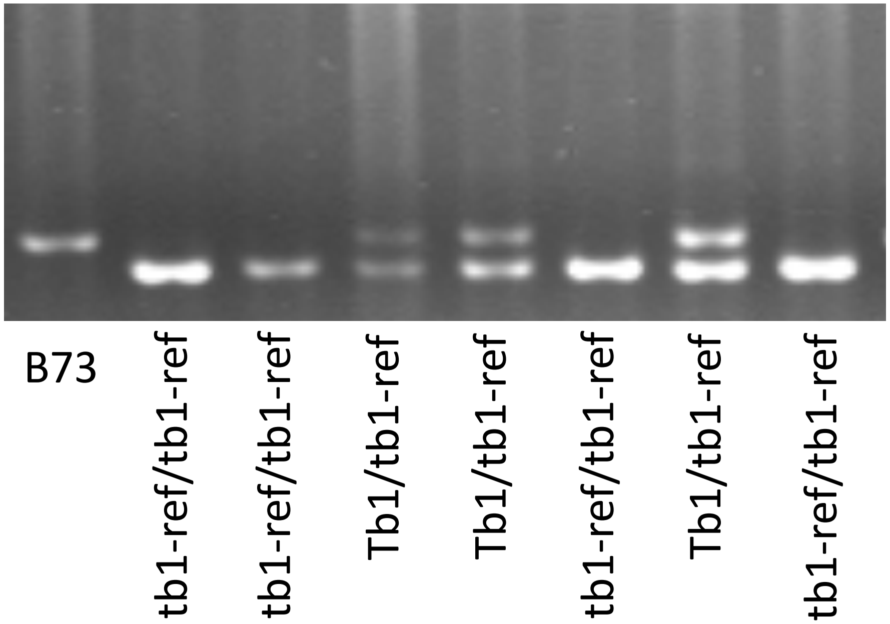


**Figure S1.** Example of *tb1-ref* allele genotyping using the umc1082 diagnostic PCR molecular marker. Shown is a 2% agarose gel performed on maize seedlings that were subsequently subjected to morphometric analysis in the greenhouse.

Figure S2

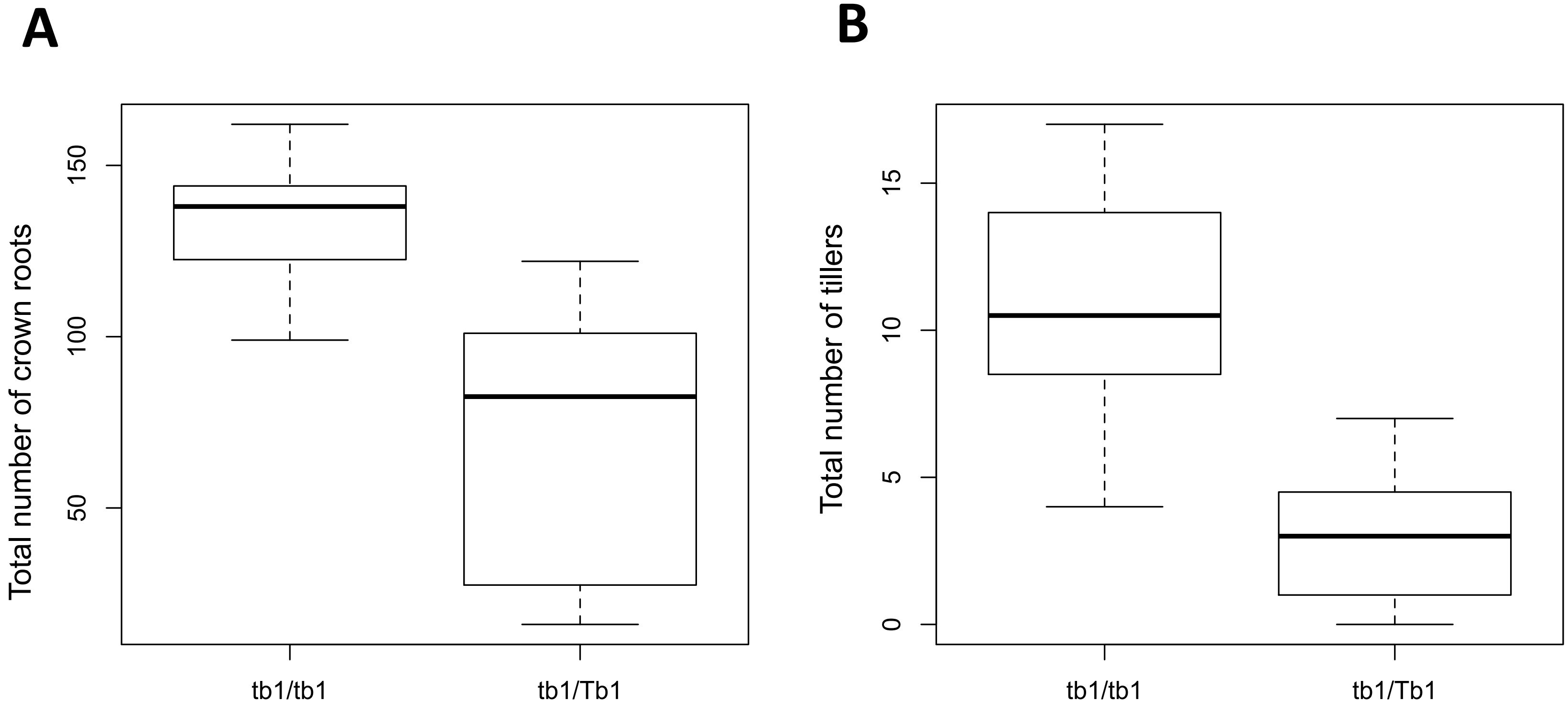


**Figure S2. Phenotypic variation within and between populations of *tb1-ref* homozygous and heterozygous plants at 35 days after transplanting for (A) the total number of crown roots per plant and (B) the total number of shoot tillers per plant.** The range of values demonstrates that the two genotypes had distinct phenotypes associated with altered *tb1-ref* allele dosage, despite hypothetical genetic modifiers that may or may not have been segregating in the background (n=12).
