# Supplementary material for: The effect of altered dosage of a mutant allele of Teosinte branched 1 (tb1-ref) on the root system of modern maize
Source: BMC Genet. 2014 Feb 14;15:23. doi: 10.1186/1471-2156-15-23 (PMC3930895; doi:10.1186/1471-2156-15-23)
Supplement: Additional file 2: Table S1. — Comparisons of tiller initiation date (days after transplanting) in tb1-ref heterozygotes (tb1/Tb1, B73 background) and homozygotes (tb1/tb1, B73 background) compared to modern maize inbred B73 and Balsas teosinte. Table S2. Comparisons of numbers of associated crown roots initiating from the base of each stem or tiller in tb1-ref heterozygotes (tb1/Tb1, B73 background) and homozygotes (tb1/tb1, B73 background) compared to modern maize inbred B73 and Balsas teosinte. [file 1471-2156-15-23-S2.doc]

**Additional file 2**

**Table S1.** Comparisons of tiller initiation date (days after transplanting) in *tb1*-*ref* heterozygotes (*tb1*/*Tb1*, B73 background) and homozygotes (*tb1/tb1*, B73 background) compared to modern maize inbred B73 and Balsas teosinte.

|  | Sequentially initiated tillers | | | | | | | | | | | | | | | | | | | | | |
| --- | --- | --- | --- | --- | --- | --- | --- | --- | --- | --- | --- | --- | --- | --- | --- | --- | --- | --- | --- | --- | --- | --- |
|  | T1 | T2 | T3 | T4 | T5 | T6 | T7 | T8 | T9 | T10 | T11 | T12 | T13 | T14 | T15 | T16 | T17 | T18 | T19 | T20 | T21 | T22 |
| *tb1/Tb1* | 20 | 25 | 25 | 30 | 35 | N/A | N/A | N/A | N/A | N/A | N/A | N/A | N/A | N/A | N/A | N/A | N/A | N/A | N/A | N/A | N/A | N/A |
| *tb1/tb1* | 15 | 15 | 20 | 20 | 20 | 25 | 25 | 25 | 25 | 30 | 30 | 35 | 35 | 35 | 35 | N/A | N/A | N/A | N/A | N/A | N/A | N/A |
| B73 | N/A | N/A | N/A | N/A | N/A | N/A | N/A | N/A | N/A | N/A | N/A | N/A | N/A | N/A | N/A | N/A | N/A | N/A | N/A | N/A | N/A | N/A |
| Teosinte | 15 | 15 | 20 | 20 | 25 | 25 | 25 | 25 | 25 | 25 | 25 | 25 | 30 | 30 | 30 | 30 | 30 | 30 | 30 | 30 | 35 | 35 |

**Notes:**

-Tiller (T1 to T22) initiation date was recorded every 5 days from transplanting to harvest at 35 days.

-Initiation dates are expressed as days after transplanting.

-Values are least square means from ANOVA ± standard errors (n=12) at 35 days after transplanting.

-T= tiller. N/A=Non applicable.

-Means within a column followed by the same letter are not significantly different at α=0.05.

**Table S2.** Comparisons of numbers of associated crown roots initiating from the base of each stem or tiller in *tb1*-*ref* heterozygotes (*tb1*/*Tb1*, B73 background) and homozygotes (*tb1/tb1*, B73 background) compared to modern maize inbred B73 and Balsas teosinte.

|  |  |  | Sequentially initiated tillers | | | | | | | | | | | | | | | | | | | | | |
| --- | --- | --- | --- | --- | --- | --- | --- | --- | --- | --- | --- | --- | --- | --- | --- | --- | --- | --- | --- | --- | --- | --- | --- | --- |
|  | Total | Main stem | T1 | T2 | T3 | T4 | T5 | T6 | T7 | T8 | T9 | T10 | T11 | T12 | T13 | T14 | T15 | T16 | T17 | T18 | T19 | T20 | T21 | T22 |
| *tb1/Tb1* | 98.4^a^ | 44.9 ^a^ | 14.8 | 16.3 | 11.1 | 8.8 | 2.5 | N/A | N/A | N/A | N/A | N/A | N/A | N/A | N/A | N/A | N/A | N/A | N/A | N/A | N/A | N/A | N/A | N/A |
| *tb1/tb1* | 130.3^b^ | 44.7 ^a^ | 14.0 | 19.2 | 11.3 | 11.2 | 4.7 | 6 | 2.3 | 2 | 1 | 3 | 5 | 1 | 3 | 1 | 2 | N/A | N/A | N/A | N/A | N/A | N/A | N/A |
| B73 | 48.8^c^ | 48.8 ^a^ | N/A | N/A | N/A | N/A | N/A | N/A | N/A | N/A | N/A | N/A | N/A | N/A | N/A | N/A | N/A | N/A | N/A | N/A | N/A | N/A | N/A | N/A |
| Teosinte | 168.3^d^ | 33.8 ^a^ | 17.0 | 20.0 | 12.0 | 12.8 | 12.2 | 7.2 | 4.3 | 3.8 | 1.5 | 4.8 | 7.0 | 2.0 | 5.5 | 5.3 | 2.0 | 3.0 | 2.8 | 2.0 | 3.7 | 2.0 | 2.0 | 2.0 |

**Notes:**

-Values are least square means from ANOVA ± standard errors (n=12) at 35 days after transplanting.

-T= tiller. N/A=Non applicable.

-Means within a column followed by the same supercript letter are not significantly different at α=0.05. Values within a column followed by distinct supercript letters indicate that the means are significantly different at α=0.05.
